# In Vitro Metabolic Study of Four Synthetic Cathinones: 4-MPD, 2-NMC, 4F-PHP and bk-EPDP

**DOI:** 10.3390/metabo12020115

**Published:** 2022-01-25

**Authors:** Ivana Gavrilović, Yunita Gelu, Vincenzo Abbate

**Affiliations:** 1 Drug Control Centre, King’s College London, Franklin Wilkins Building, 150 Stamford Street, London SE1 9NH, UK; ivana.gavrilovic@kcl.ac.uk; 2 Department of Analytical, Environmental and Forensic Sciences, King’s College London, 150 Stamford Street, London SE1 9NH, UK; yunita.gelu@kcl.ac.uk

**Keywords:** NPS, 4-MPD, 2-NMC, 4F-PHP, bk-EPDP, in vitro metabolism, LC–HRMS

## Abstract

The use of illicit drugs is exceedingly prevalent in society, and several of them can be illegally purchased from the internet. This occurrence is particularly augmented by the rapid emergence of novel psychoactive substances (NPS), which are sold and distributed as “legal highs”. Amongst NPS, the class of synthetic cathinones represents stimulant substances exhibiting similar effects to amphetamine and its derivatives. Despite potentially being less psychoactive than amphetamine, synthetic cathinones are harmful substances for humans, and little or no information is available regarding their pharmacology and toxicology. The present study investigated the in vitro metabolism and metabolites of four recent synthetic cathinones, namely, 1-(4-methylphenyl)-2-(methylamino)-pentanone (4-MPD), 1-(4-methylphenyl)-2-dimethylamino-propanone (2-NMC), 1-(4-fluorophenyl)-2-(pyrrolidin-1-yl-hexanone (4F-PHP) and 1-(1,3-benzodioxol-5-yl)-2-(ethylamino)-1-pentanone (bk-EPDP). Our in vitro metabolism study resulted in 24 identified metabolites, including both phase I and phase II metabolites. All metabolites were detected and identified using liquid chromatography–high-resolution mass spectrometry and may serve as additional markers of abuse of these NPS in toxicological analyses.

## 1. Introduction

The use of illicit drugs is exceedingly prevalent in society, and recently emerging designer drugs can be illegally purchased via the internet. Novel psychoactive substances (NPS) are illicit drugs synthesised with the purpose of mimicking conventionally established recreational drugs. Many subclasses of NPS have been recognised, including stimulants, synthetic cannabinoids, hallucinogens and synthetic opioids [1]. According to the United Nations World Drug Report, the manufacture of amphetamine-like stimulants seems to be the most dominant, and seizures of these drugs increased four-fold between 2009 and 2018. There are reports that, in some countries, methamphetamine seizures decreased because mephedrone and its derivatives became more prominent [2]. This shift in the NPS market to synthetic cathinones seems to be a trend that is widespread in global society. Although some of these compounds have been scheduled as controlled substances, most of them are still sold and distributed as “legal highs”. Furthermore, in order to avoid legal restrictions, some of these newly emerged drugs are mislabelled as chemicals used for research purposes and not for human consumption [3]. 

Cathinone is the main active ingredient in the khat shrub. Due to the similarity in structure to amphetamine and amphetamine derivatives, cathinone is widely used for its stimulant effects [4]. Cathinone has served as a template drug for the synthesis of a wide range of novel and very potent synthetic cathinones (SCt), such as mephedrone, methcathinone and methylone, which also exhibit amphetamine-like effects [4,5]. 

**Figure 1:**
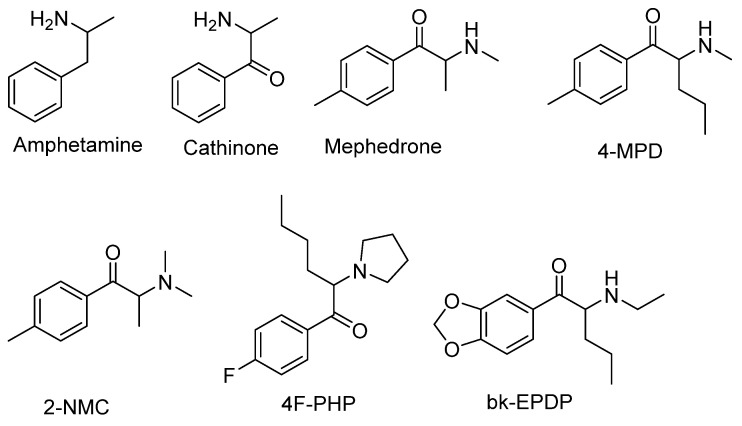
Chemical structures of amphetamine, cathinone, mephedrone and synthetic cathinone derivatives investigated in this study.

The synthesis of NPS, including SCt, is a relatively quick and straightforward process, and therefore, NPS are constantly emerging on the market to potentially avoid legislative controls. For instance, Apirakkan et al. recently reported the identification and analytical characterisation of three emerging synthetic cathinones, namely: 1-(4-methylphenyl)-2-(methylamino)-pentanone (4-MPD), 1-(4-fluorophenyl)-2-(pyrrolidin-1-yl-hexanone (4F-PHP) and 1-(1,3-benzodioxol-5-yl)-2-(ethylamino)-1-pentanone (bk-EPDP) [6]. Figure 1 illustrates the structures of amphetamine, cathinone and selected SCt derivatives, including those investigated in this study.

Information about the toxic effects of SCt and their detection in biological matrices (e.g., urine and blood) does not reach the scientific community as quickly as they emerge on the market due to the straightforward production and subsequent availability of numerous analogues with similar substituents. For instance, Majchrzak et al. reviewed SCt that appeared on the illegal drug market from 2014 to 2017 and discovered more than 30 cathinone derivatives [7]. When detecting the presence of SCt in human matrices, the parent drug may not always be detected in the biological matrix (e.g., urine, saliva, hair and blood) due to its relatively short half-life. Studies on human urine samples also showed that only a small amount of SCt was excreted as the parent drug, whereas the metabolised form was present in a larger amount [8], suggesting that targeting SCt metabolites in toxicological investigations may extend their window of detection. Conducting administration studies of emerging NPS with healthy human volunteers carries imposed health risks. Hence, it is very hard to obtain ethical permission to conduct studies of such kind. However, elucidating the metabolic pathways of these drugs and identifying the metabolite structures provide important information that can be useful in analytical toxicology and forensic science [9,10,11]. Investigating NPS metabolites may also be useful in assisting forensic investigations, such as drug-facilitated sexual assault or drug driving, to determine whether the involved people were under the influence of certain substances when the incident occurred [12,13]. Considering the existing difficulties of investigating the in vivo metabolism of NPS, this research employed the use of human liver microsomes and S9 fractions to elucidate the in vitro metabolism of four emerging SCt, namely, 1-(4-methylphenyl)-2-(methylamino)-pentanone (4-MPD), 1-(4-methylphenyl)-2-dimethylamino-propanone (2-NMC), 1-(4-fluorophenyl)-2-(pyrrolidin-1-yl-hexanone (4F-PHP) and 1-(1,3-benzodioxol-5-yl)-2-(ethylamino)-1-pentanone (bk-EPDP). We report the detection and identification of their metabolites by liquid chromatography–high-resolution mass spectrometry (LC–HRMS). This analytical technique is suitable for the identification of SCt in any forensic toxicology laboratory due to the provision of accurate mass measurements and the possibility of retrospective data analysis [14]. 

## 2. Results

The in vitro metabolic reactions using HLM and S9 were quenched with acetonitrile after 6 h of incubation, and the obtained metabolites of 4-MPD, 2-NMC, 4F-PHP and bk-EPDP were analysed by LC–HRMS. 

Several metabolites of each SCt were identified from the full-scan data when extracting the accurate protonated metabolite mass within a narrow 5 ppm extraction window. A blank sample containing only reagents and enzymes but no SCt was also investigated to confirm that peaks representing the identified metabolites were specific to the metabolism study. No interfering peaks were detected in any of the blank samples (data not shown). In total, 24 phase I and II metabolites were identified in our study, as summarised in Table 1 below and Table S1 (supplementary material). Metabolite structure elucidation was conducted via targeted MS^2^ experiments. 

## 3. Discussion

Due to the general structural similarities, the identification of metabolites, their fragmentation patterns and their metabolic pathways in our study were compared with previously studied SCt, such as mephedrone, 4-methylethcathinone (4-MEC), pentylone, methylone, 1-phenyl-2-(pyrrolidin-1-yl)pentan-1-one (α-PVP) and 3,4-methylenedioxypyrovalerone (MDPV) [15,16,17,18,19,20].

### 3.1. 4-MPD In Vitro Metabolism

**Figure 2:**
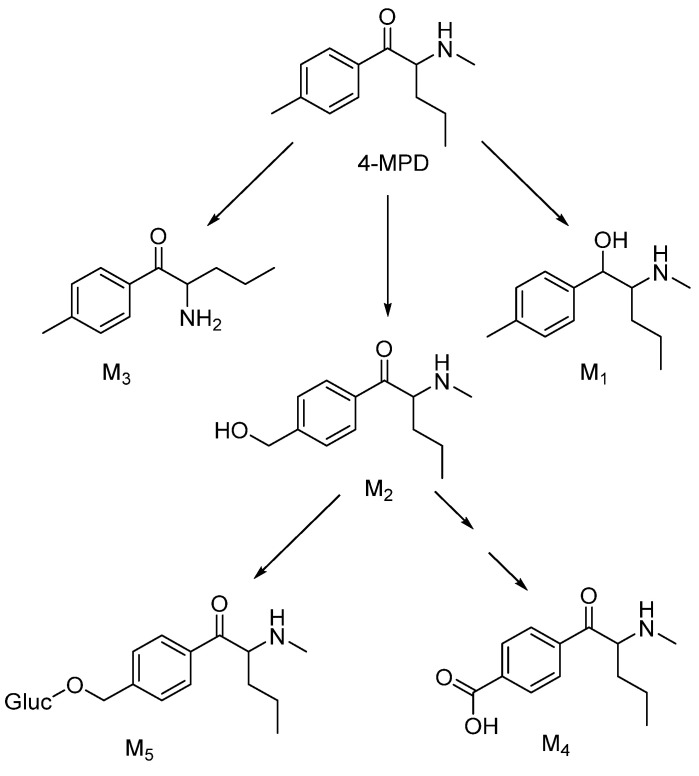
Proposed metabolic pathway for 4-MPD (metabolite numbering in accordance with Table 1).

In our study, four phase I and one phase II glucuronide metabolites of 4-MPD (M1–M5, Table 1) were detected, with a mono-hydroxylated metabolite (M2) being the most abundant species. Our in vitro results are in accordance with some findings in previous in vitro studies on 4-methyl-N-ethyl-cathinone and mephedrone [16,21]. These studies proposed the following metabolic pathways: reduction of the oxo group and N-dealkylation and hydroxylation of the 4-methyl group to the corresponding 4-carboxy metabolite. Glucuronides were formed from hydroxyl metabolites. Our observed metabolic reactions include reduction of the oxo group (M1), N-demethylation (M3) and 4′-methyl hydroxylation (M2), followed by further oxidation, namely, carboxylation (M4). The glucuronide metabolite identified in our study was obtained in the reaction of a hydroxyl metabolite with glucuronic acid, which is also consistent with a mephedrone phase II metabolite detected in human urine [22]. Based on the identified metabolites, a metabolic pathway for 4-MPD was postulated, as shown in Figure 2.

Due to the large amount of the drug incubated with the enzymes, the presence of unmetabolised 4-MPD was confirmed by identifying its protonated precursor ion at *m*/*z* 206.1539 and some major product ions, such as *m*/*z* 188.1434 (Δppm = −0.1053, water loss), *m*/*z* 175.1118 (Δppm = 0.0784, CH_5_N loss), *m*/*z* 146.0964 (Δppm = −0.1433, C_3_H_8_O loss), *m*/*z* 133.0646 (Δppm = −1.5107, C_5_H_13_N loss) and *m*/*z* 105.0699 (Δppm = −0.0654, C_5_H_11_ON loss). The MS^2^ spectrum (Figure S1) showed that *m*/*z* 188.1434 was the most abundant species, corresponding to the loss of a water molecule. This fragmentation pattern was also compared to that of mephedrone from a previous study in which *m*/*z* 119, suggested to be C_8_H_7_O^+^, was also identified [23]. Based on the postulated elemental composition and the exact mass, it can be concluded that *m*/*z* 119.0491 in our study corresponds to the same molecular formula and structure of those found for mephedrone.

The M1 metabolite was formed from the reduction of the oxo functional group in the β position (β-keto reduction), yielding a peak at *m*/*z* 208.1694. The M1 fragmentation pattern (Figures S2 and S3) shared some similarities to that of the 4-MPD parent compound, as evidenced by the presence of a product ion at *m*/*z* 105.0697 (Δppm = −1.4451) and the production of the most abundant species corresponding to a common loss of a water molecule observed at *m*/*z* 190.1589 (Δppm = −0.4767). A slightly different fragment from the parent compound (*m*/*z* 175.1118) was also observed at *m*/*z* 177.1273 (Δppm = −0.6670). A mass difference of 2.0155 between these observed fragments further indicated the conversion of the β-keto moiety into a hydroxyl group.

**Figure 3:**
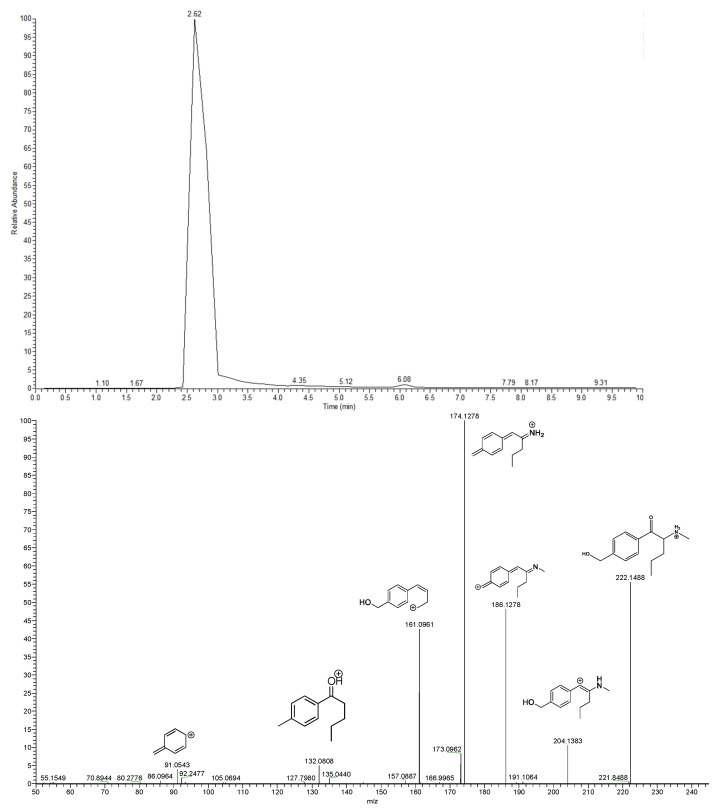
Extracted ion chromatogram and MS^2^ spectra including the proposed fragmentation pattern of 4-MPD M2.

The M2 metabolite (accurate *m*/*z* 222.1488, Δppm = −0.3701) (Figure 3 and Figure S4) was the most abundantly detected 4-MPD metabolite in our in vitro system and was formed due to oxidative introduction of a hydroxyl group on the tolyl moiety of 4-MPD. This position of hydroxylation was also postulated because of the subsequent identification of an M4 carboxylic acid metabolite, the oxidised form of a hydroxy-tolyl metabolite [15,16]. The loss of two water molecules was deduced from the 4-MPD M2 fragmentation pattern (Figure 3 and Figure S4). Such losses were firstly indicated by a fragment ion from the M2 protonated precursor ion to *m*/*z* 204.1383 (Δppm = −0.1511) due to the primary loss of water, followed by a fragment at *m*/*z* 186.1278 (Δppm = 0.2741) due to the loss of a further water molecule, followed by a proposed intramolecular rearrangement. Contrary to the parent molecule and M1 metabolite, the most abundant species in the M2 fragmentation pattern is the loss of two water molecules followed by N-demethylation, observed at *m*/*z* 174.1278 (Δppm = 0.3806).

Enzymatic removal of a methyl group from the nitrogen atom resulted in the formation of the M3 metabolite, as evidenced by the species detected at *m*/*z* 192.1383 (data not presented). For the M3 metabolite, the loss of a water molecule was observed as the most abundant fragment in the MS^2^ spectra, leading to *m*/*z* 174.1277 (Δppm = 0.0301). The product ions that were observed for 4-MPD were also observed for the M3 metabolite, including *m*/*z* 119.0492 (Δppm = 0.7009, C_4_H_11_N loss) and *m*/*z* 105.0699 (Δppm = 0.0072, C_4_H_9_NO loss). These fragments indicate the unchanged general structure of the M3 metabolite compared to its parent molecule, with the exception of N-demethylation.

As previously indicated, M4 was formed through the metabolic oxidation of the hydroxy-tolyl metabolite M2 and identified at *m*/*z* 236.1279. Due to this metabolic pathway and since both compounds share structural similarities in the remainder of the molecule, the fragmentation pattern of the M4 metabolite (data not presented) is comparable to that of the M2 metabolite. This similarity was evidenced by the subsequent loss pattern of two water molecules, which seems quite favourable for this metabolite, as two fragments, *m*/*z* 218.1171 (Δppm = −1.8960) and *m*/*z* 200.1068 (Δppm= −1.0475), were observed. The same fragment observed for M2 at *m*/*z* 174.1275 was also observed for the M4 metabolite. The only difference was that instead of experiencing the loss of two water molecules and N-demethylation, the fragment observed at *m*/*z* 174.1275 for M4 (Δppm = −1.1967) was the result of the loss of three water molecules followed by N-demethylation. Manier et al. investigated in vitro metabolism of 4-MPD using HLM with a similar experimental setting. They identified what we describe as M1 and M4 and reported metabolites formed by 4-MPD hydroxylation of the alkyl chain and N-oxidation, which we did not identify [24]. Our work offers further insights into phase II metabolism in comparison to their work. 

**Figure 4:**
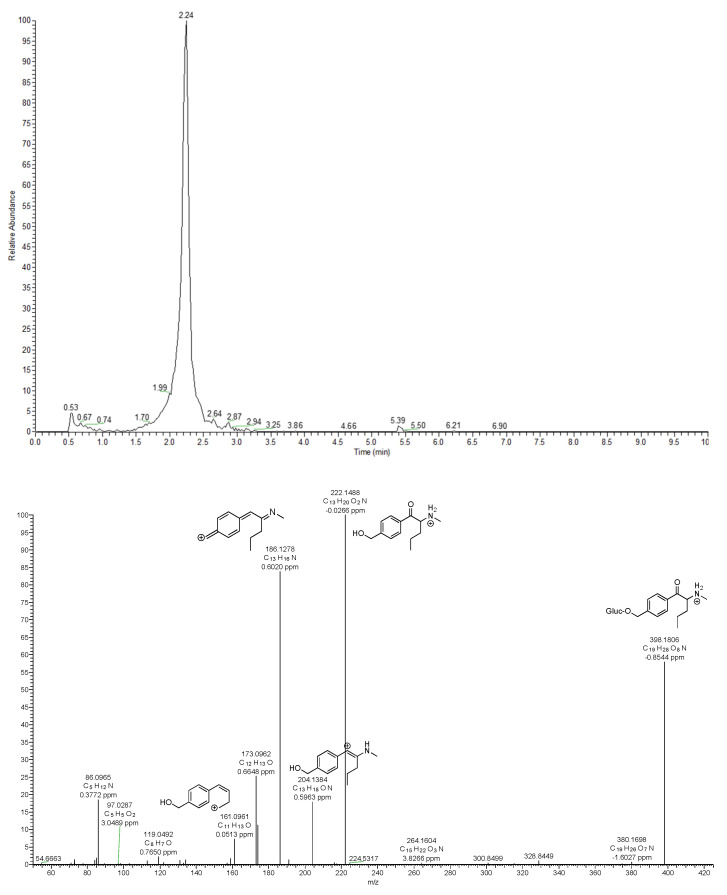
Extracted ion chromatogram and MS^2^ of M5 glucuronide, phase II metabolite of 4-MPD.

M5 glucuronide was the only phase II metabolite identified for 4-MPD and observed at *m*/*z* 398.1806 (Δppm = −0.8544) (Figure 4). This metabolite was further confirmed to be the glucuronide form of the M2 metabolite, as the fragmentation pattern showed the presence of *m*/*z* 222.1488, corresponding to the protonated mass of the M2 metabolite after the loss of glucuronic acid (C_6_H_10_O_7_ loss). In this study, no other phase II metabolites formed from 4-MPD were detected. A possible explanation for the regioselective glucuronidation could be that the position of the OH group in M2 is more accessible compared to the hydroxyl position in M1.

### 3.2. 2-NMC In Vitro Metabolism

**Figure 5:**
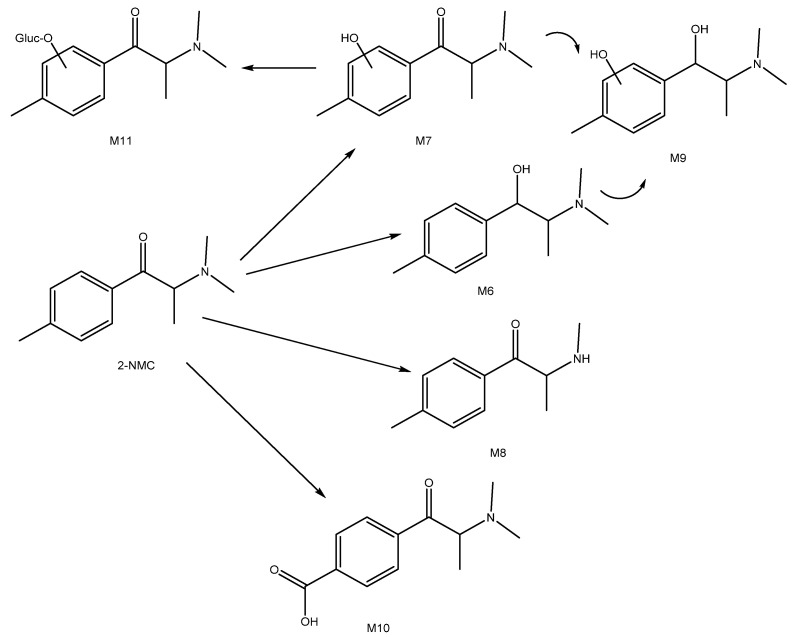
Proposed metabolic pathway for 2-NMC (metabolite numbering in accordance with Table 1).

Generally, similar metabolite species identified for 4-MPD were identified for 2-NMC (Figure 5, M6–M11, Table 1 and Table S1).

The most abundant metabolite was M6, where the parent drug underwent β-oxo group reduction. The remaining unmetabolised 2-NMC was confirmed by identifying its protonated precursor ion at *m*/*z* 192.1383 (Δppm = −0.1606) (data not presented). Even though its general structure is somewhat similar to that of 4-MPD, the 2-NMC fragmentation pattern showed slightly different product ions. The loss of a water molecule does not seem to be favoured in 2-NMC fragmentation. Furthermore, instead of producing a fragment at *m*/*z* 146, there was a fragment observed at *m*/*z* 147.0804 (Δppm = −0.0847, C_2_H_7_N loss), in which the keto/oxo functional group is intact together with the benzene ring and methyl side chain. This particular fragment was also identified in a previous study of mephedrone, where this fragment was suggested to possess an elemental composition of C_10_H_11_O^+^ [23]. Based on their accurate masses, it can be concluded that these two fragments represent the same structure and elemental composition. Another fragment was observed at *m*/*z* 119.0855 (Δppm = 0.1803), representing the loss of C_3_H_7_ON.

**Figure 6:**
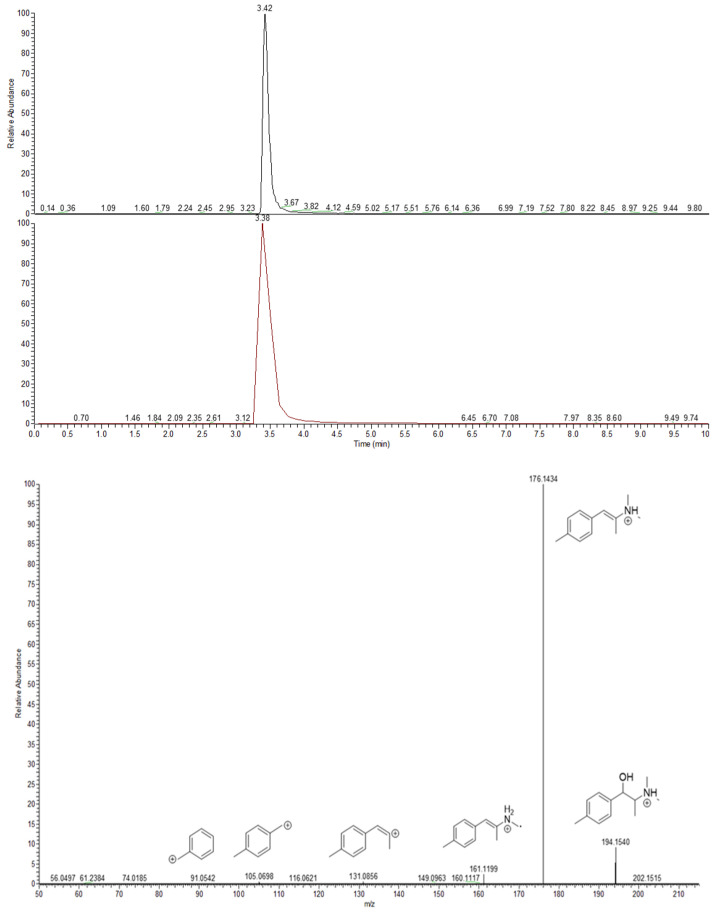
From top to bottom: total ion chromatogram (1.7 e9 counts), extracted ion chromatogram (5.4 e7 counts) and MS^2^ spectra with proposed fragmentation pattern of M6, the most abundant in vitro metabolite of 2-NMC.

The M6 metabolite was formed through a reduction of the 2-NMC oxo functional group, leading to a shift from the 2-NMC protonated precursor ion to *m*/*z* 194.1540 (Δppm = 0.1836). Even though the structure of this metabolite only differs in the presence of a hydroxyl group instead of oxo in the parent molecule, its fragmentation pattern is different. Unlike 2-NMC, the loss of a water molecule was the most abundant fragment in the M6 metabolite fragmentation pattern, as indicated by a fragment ion at *m*/*z* 176.1434 (Δppm = −0.0259). This indicated that, contrary to the parent molecule, the M6 metabolite contained a secondary hydroxyl group that was more readily released as a water molecule [19]. Other fragments detected were *m*/*z* 161.1199 (Δppm = 0.2997) due to N-demethylation, 131.0856 (Δppm = 0.4548, C_2_H_9_NO loss), 105.0698 (Δppm = −0.7916, C_4_H_11_NO loss) and *m*/*z* 91.0542 (Δppm = −0.3030), resulting from hydroxyl group-α-cleavage of the parent compound. The full-scan extracted ion chromatogram and MS^2^ spectrum of this metabolite and its postulated fragmentation pattern are shown in Figure 6 and Figure S5.

The fragmentation pattern of the M7 metabolite (*m*/*z* 208.1332, Δppm = −0.0546) demonstrated the loss of water, as a fragment ion at *m*/*z* 190.1229 (Δppm = 1.4944) was detected (Figure S6). A fragment ion observed for the parent compound (*m*/*z* 147.0804) with a shift of 2.0158 to *m*/*z* 149.0962 (Δppm = 0.5671) showed that the keto/oxo functional group of the molecule was still intact together with the benzene ring and methyl side chain. Fragment ions were observed at *m*/*z* 190.1229 (water loss due to β-keto cleavage) (Δppm = 1.4944), 149.0962 (Δppm = 0.5671) and 133.0648 (Δppm = 0.2094), all retaining the hydroxylated group bound to the aromatic moiety, and *m*/*z* 119.0492 (Δppm = 0.4446) following an intramolecular rearrangement. The results of the MS^2^ fragmentation experiment on the M7 metabolite did not favour the pattern in which its keto/oxo functional group was released from the molecule. The data from the MS^2^ extracted ion chromatograms showed the presence of an additional peak at 1.10 min. This finding was then considered to be an indication of the presence of another isomer of the M7 metabolite that differs in the position of hydroxylation, despite the significant difference in retention times [25]. However, this hypothesis can only be tested when analysing their reference materials. 

The M8 metabolite was produced through a metabolic reaction that turned the tertiary amine into a secondary amine via removal of a methyl group, leading to a protonated precursor ion at *m*/*z* 178.1226 (Δppm = −0.0325). Of the MS^2^ product ions of this metabolite (data not presented), the most abundant product ion was at *m*/*z* 160.1121 (Δppm = −0.0966), corresponding to the loss of water. It was suggested that the loss of water was followed by the rearrangement of the M8 structure and formation of a cyclic amine attached to the cyclohexane ring [17]. This rearrangement led to the presence of a product ion at *m*/*z* 145.0886 (Δppm = 0.1524).

The M9 metabolite was produced from a combination of two metabolic reactions, which were the reduction of the β-keto group and (aromatic) hydroxylation, leading to a protonated precursor ion observed at *m*/*z* 210.1488 (Δppm = −0.2460). The fragmentation pattern (Figure S7) showed a product ion at *m*/*z* 192.1383 (Δppm = −0.0017) consistent with the loss of a molecule of water, most likely from the β-keto group. Further cleavage of the N,N-dimethyl group led to a product ion observed at *m*/*z* 149.0961 (Δppm = 0.2601), followed by the second hydroxyl group loss and intramolecular rearrangement, leading to a fragment at observed *m*/*z* 131.0856 (Δppm = 0.2220).

Metabolite M10 was detected in the S9 fraction (*m*/*z* 222.1126, Δppm = 0.5615). As observed for 4-MPD phase II metabolites, M11 glucuronide was the only glucuronide metabolite identified for 2-NMC (*m*/*z* 384.1645, Δppm = −2.1708). It was assumed that metabolite M11 was not formed in the reaction of glucuronic acid with M6, the most abundant 2-NMC metabolite, but with M7, the mono-hydroxy metabolite. It is possible that this preference was caused by a steric effect present on the molecule of the M6 metabolite. This steric effect possibly occurs from three methyl groups attached to the nitrogen atom, which potentially hinders the glucuronidation reaction site. Even though M11 glucuronide is present in a rather low amount, the fragments of initial phase I metabolites (M7, *m*/*z* 208.1330, Δppm = −0.9344) can be identified as a result of glucuronic acid loss after fragmentation.

Lopes et al. investigated in vitro phase I and phase II glucuronidation metabolism of various synthetic cathinones, including 2-NMC [26]. Cathinones were incubated with cofactors and enzymes (human and rat liver microsomes) and subsequently analysed by LC–HRMS. Apparently, 4-NMC phase I metabolites were not identified, but phase II glucuronide was identified. Glucuronidation occurs after demethylation (2-NMC loses one n-methyl group, the M8 metabolite in our work) and hydroxylation at the remaining N-methyl group. We identified 2-NMC hydroxy metabolites, but we propose a hydroxylation reaction on the benzene ring. 

### 3.3. 4F-PHP In Vitro Metabolism

**Figure 7:**
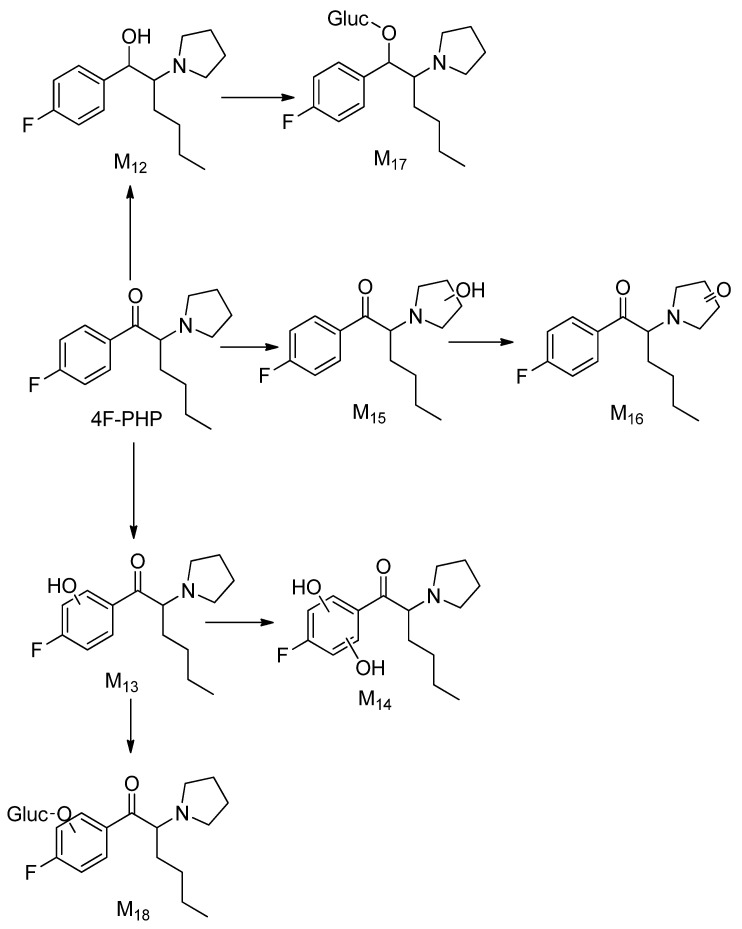
Proposed metabolic pathway of 4F-PHP (metabolite numbering according to Table 1).

A total of seven 4F-PHP phase I (M12–M17) and two phase II (M17 and M18 glucuronides) metabolites were detected and identified. The 4F-PHP metabolic pathway (Figure 7) was found to mirror the reported in vitro metabolism of α-PVP, pyrovalerone (MPVP) and 4′-methyl-α-pyrrolidinohexiophenone (MPHP) [18,19]. Here, the oxo metabolite (M16) was found in a rather lower amount compared to the abundance of other metabolites. Our study found that the metabolite with a reduced β-keto group (M12) was the most abundant species.

The parent compound was identified based on its protonated precursor ion showing the protonated molecular mass (*m*/*z* 264.1754, Δppm = −1.4140). Low-abundance fragments were produced from the parent compound MS^2^ spectrum (Figure S8). The two most distinct product ions were observed at *m*/*z* 193.1024 (Δppm = 0.1871), corresponding to the loss of the pyrrolidine ring, and at *m*/*z* 109.0448 (Δppm = −0.1174), indicating β-cleavage followed by the loss of a water molecule. 

**Figure 8:**
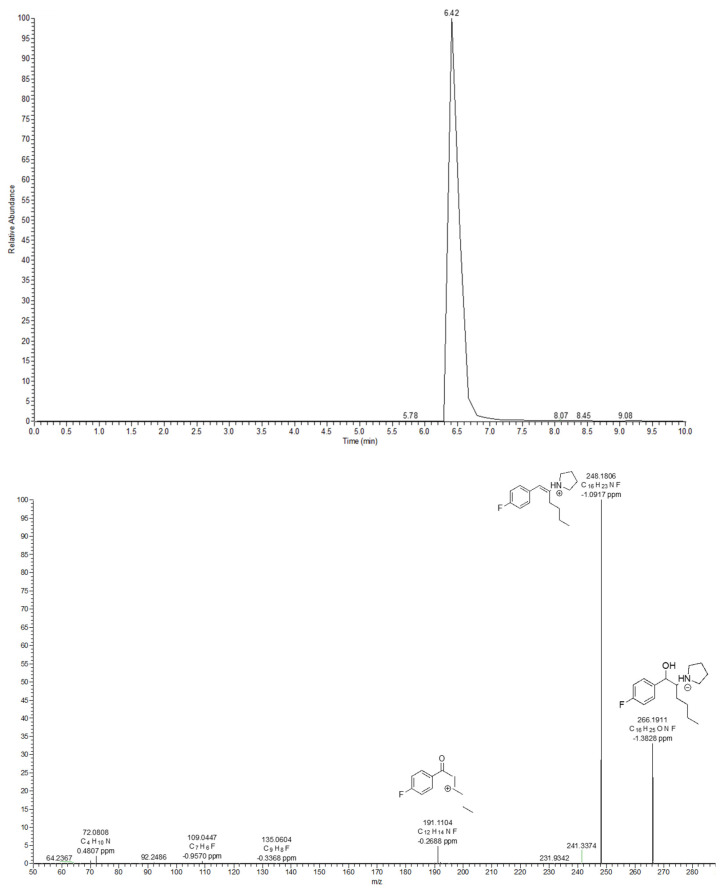
4F-PHP M12 extracted ion chromatogram and MS^2^ spectra.

The M12 metabolite was formed through a reduction of the keto/oxo group, leading to *m*/*z* 266.1911 (Δppm = −1.3828). Unlike the parent compound, the loss of a water molecule seems the most favoured for M12, as the most dominant ion was observed at *m*/*z* 248.1806 (Δppm = −1.0917) (Figure 8).

Both metabolites M13 and M15 were formed due to a metabolic hydroxylation at different positions and resulted in a mixture of positional isomers. Therefore, these metabolites are isomeric and thus isobaric species, exhibiting identical calculated protonated masses. Both M13 and M15 protonated precursor ions were observed at *m*/*z* 280.1703 (data not presented). Both species share similar fragmentation patterns with the parent compound. We propose that the M13 metabolite is formed by phenyl hydroxylation, which occurred at two different positions, since two peaks were detected at 3.39 min and 3.59 min. The product ion observed at *m*/*z* 123.0239 (Δppm = −1.7265) indicated the presence of a hydroxyl group attached to the benzene ring in M13. One characteristic fragment for M15 (Rt = 5.31 min) was observed at *m*/*z* 156.1383 (Δppm = −0.1976), demonstrating that the hydroxylation occurred on the pyrrolidine ring. It is assumed that the hydroxylation occurring on the benzene ring (M13) produces more polar metabolites compared to the one hydroxylated on the pyrrolidine ring, hence the different elution times.

The M14 metabolite was formed through two subsequent hydroxylations of the parent molecule, leading to *m*/*z* 296.1652 (Δppm = −1.4388). This type of dihydroxy metabolite was not observed on the other three synthetic cathinones analysed in this study. Although the positions of the two hydroxyl groups cannot be confidently deduced, they are likely to reside on the aromatic moiety. Metabolic oxidation of the hydroxyl group on the M15 metabolite resulted in the formation of the M16 metabolite, leading to a protonated precursor ion at *m*/*z* 278.1548 (Δppm = −1.5119). A similar characteristic fragment (*m*/*z* 156) observed for the M15 metabolite was also identified with a shift of 2.0158 to *m*/*z* 154.1224 (Δppm = −1.7207). This difference further confirmed the oxidative biotransformation of the hydroxyl group attached to the pyrrolidine ring into a carbonyl group.

M17 and M18 glucuronides were formed through conjugation reactions between M12 and M13 metabolites, respectively, with glucuronic acid. These two metabolites were identified as the only 4F-PHP phase II metabolites. The protonated precursor ions were observed at *m*/*z* 442.2234 (Δppm = −0.4496) for M17 and *m*/*z* 456.2025 (Δppm = −0.8065) for M18. Both glucuronidated metabolites product ion scans (data not presented) displayed the typical loss of glucuronic acid, followed by the loss of a water molecule. The M18 peak appeared as a doublet, which may further prove our claim that two M13 metabolites detected were positional isomers. Two close M18 Rt (4.67 and 4.80 min) clearly highlighted the differential position of the sugar moiety attached to the phase I metabolite.

Carlier et al. investigated the phase I metabolic profile of pyrrolidinyl SCts, α-PHP and 4F-α-PVP, using pooled human hepatocyte incubations and LC–HRMS analysis [27]. Markers for both cathinones were suggested, and 4F-α-PVP metabolism was described for the first time, although further experiments with suitable synthesised analytical standards are needed to confirm the findings. 4F-α-PVP is structurally related to 4F-PHP, the cathinone investigated in our study. The major 4F-α-PVP metabolic reactions included reduction of the β-keto group and oxidation of the pyrrolidinyl ring, which mirror metabolites detected in this study (M12 and M16). Pyrrolidinyl dihydroxylation was a major 4F-α-PVP metabolic transformation. In our work, dihydroxylation occurred on the phenyl ring. Carlier et al. reported that dealkylation to the primary amine and alkyl hydroxylation were identified as minor phase I metabolites in both α-PHP and 4F-α-PVP, but we did not identify any similar metabolites for 4F-PHP.

Wagmann et al. published a study describing the toxicokinetics of synthetic cathinones, including 4F-PHP [28]. Urine and blood samples were collected from a male who was admitted into the hospital due to aggressive behaviour and uncontrolled moves. The analysis was conducted by LC–HRMS and gas chromatography–mass spectrometry (GC–MS). 4F-PHP was identified in patients’ blood and urine, and the investigation was extended to elucidating and identifying any metabolites present. In vitro drug metabolism was performed with the S9 fraction, and identification of specific monooxygenases taking part in metabolic reactions was performed with HLM. Wagmann et al.’s study provided comprehensive information about phase I and II metabolic reactions, which led them to calculate the plasma concentrations of metabolites and propose metabolites for target screening. The metabolite matching our M16 was recommended by Wagmann et al. as a screening target for urine analysis.

In another detailed and informative study, the same author investigated the in vitro metabolism of 4F-PHP after incubations with HepaRG cells and zebrafish larvae and LC–HRMS analysis [29]. Clearly, our metabolites M12, M13, M15, M17 and M18 match their findings. 

### 3.4. bk-EPDP In Vitro Metabolism

**Figure 9:**
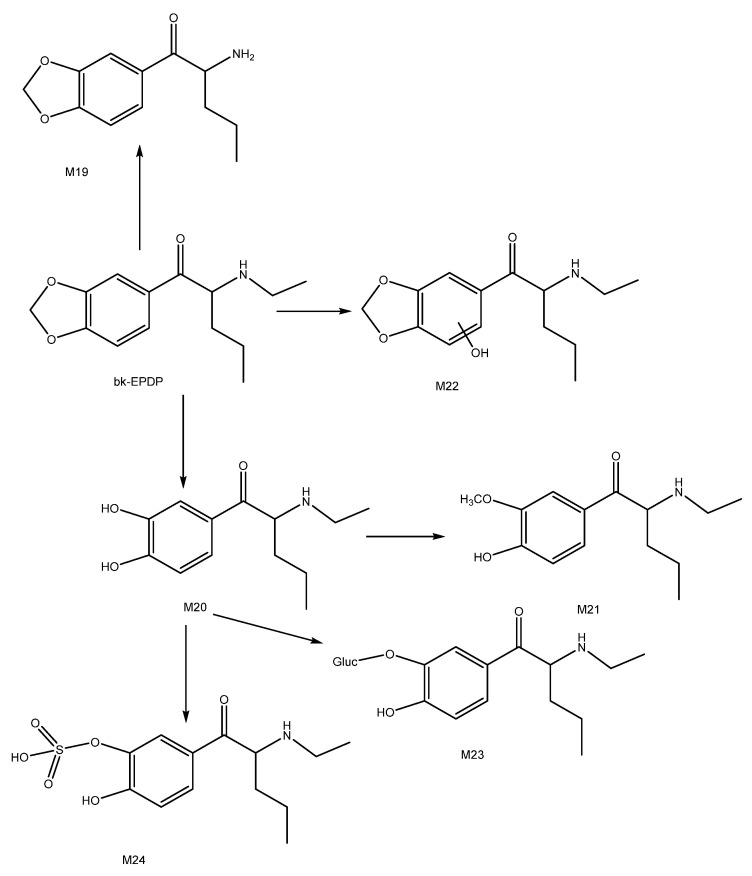
Proposed metabolic pathway of bk-EPDP (metabolite numbering according to Table 1).

After analysing bk-EPDP HLM and S9 incubates, six metabolites in total were identified, of which three phase II metabolites, namely, two glucuronides (M19 and M23) and a sulphate (M24), were identified (Figure 9). 

The phase I metabolites identified in our study are in accordance with some of the metabolites resulting from in vitro studies of structurally similar cathinones, such as MDPV, methylone, penthylone and ethylone [17,19,30,31]. None of these in vitro studies, however, reported findings on glucuronide or sulphate metabolites. Previous studies also suggested that the major metabolic pathways for synthetic cathinones endowed with a methylenedioxy ring in their structure were the hydrolysis of the methylenedioxy ring (demethylenation) followed by O-methylation. These findings are consistent with the observed results in our study, since metabolite M20 was identified as the most abundant metabolite, followed by M21.

The bk-EPDP parent compound was identified based on its protonated precursor ion, which was observed at *m*/*z* 250.1435 (Δppm = −1.1658 ppm). Based on the MS^2^ product ion spectra, it was obvious that the fragmentation pattern favoured the loss of a water molecule. This was demonstrated by the fragments observed at *m*/*z* 232.1330 (Δppm = −0.8378 ppm) (data not presented).

The M19 protonated precursor ion was observed at *m*/*z* 222.1124 (Δppm = −0.4002), which is consistent with the loss of a methyl group from the parent compound. Unlike the parent molecule, the loss of a water molecule is less favoured for M19. Instead, a fragment observed at *m*/*z* 174.0913 (Δppm = −0.0960) corresponding to the loss of one water molecule followed by CH_4_O was seen as the most abundant one (data not presented). 

**Figure 10:**
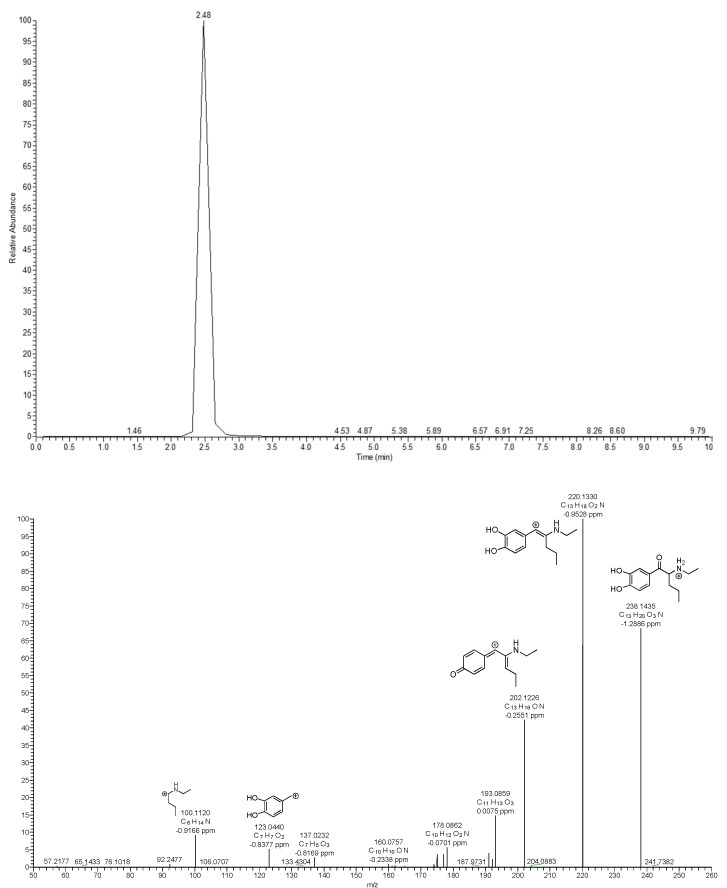
Extracted ion chromatogram and MS^2^ spectra with proposed fragmentation pattern for M20, the most abundant metabolite of bk-EPDP.

The M20 (*m*/*z* 238.1435, Δppm = −1.2886) metabolite was formed by a hydrolysis reaction on the pyrrolidine ring, which resulted in the formation of a catechol. Due to the presence of two hydroxyl groups attached to the benzene ring, the loss of more than one water molecule was expected in the M20 product ion spectrum (Figure 10). This is supported by fragments observed at *m*/*z* 220.1330 (Δppm = −0.9528) and *m*/*z* 202.1226 (Δppm = −0.2551), which is consistent with the loss of one water molecule followed by a further water loss and intramolecular rearrangement, and two water molecules, respectively. Other fragments were also observed at *m*/*z* 193.0859 (Δppm = 0.0075, C_2_H_3_N^+^ loss) and *m*/*z* 123.0440 (Δppm = −0.8377, C_6_H_13_NO^+^ loss). A similar metabolic reaction was confirmed in an in vivo study on a structurally similar compound, 3′,4′-methylenedioxy-α-pyrrolidinobutyrophenone (MDPBP) [31]. Finally, M20 was found to undergo methylation, leading to the M21 metabolite. Our MS^2^ suggests that two isomeric O-methoxy metabolites were formed. Two peaks with retention times of 2.69 min (*m*/*z* 252.1591, Δppm = −0.3167) and 4.23 min (*m*/*z* 252.1595, Δppm = 0.1106) were detected. The peak eluting at 4.23 min was about five times more abundant than the peak at 2.69 min. 

M22 was produced through a metabolic addition of hydroxyl group. Our data suggest that the hydroxylation occurs on the aromatic ring and that a mix of two isomers was formed. Lastly, glucuronide (M23) and sulphate (M24) phase II metabolites were also identified in bk-EPDP incubates. Both phase II metabolites originated from the most abundant phase I metabolite, M20. A conjugation reaction between glucuronic acid and M20 resulted in a protonated precursor ion observed at *m*/*z* 414.1754 (Δppm = −1.0691), while M20 conjugation with PAPS resulted in the sulphate phase II metabolite being detected in the S9 fraction at *m*/*z* 318.0997 (Δppm= −2.6817). A protonated molecule of the M20 metabolite after the loss of glucuronic acid and sulphate was identified on the MS^2^ spectra of both metabolites (data not presented).

Bk-EPDP was part of the previously described study by Wagmann et al. [28]. They proposed two glucuronides as screening targets, whose aglycone structures match metabolites M21 and M23 in our study. Our M19, M22 and M23 metabolites were identified in a similar in vitro study utilising HepaRG cells and zebrafish larvae [29]. 

## 4. Materials and Methods

### 4.1. Chemicals

All SCts utilised in this study were kindly provided by TicTac Communications (London, UK) and were test-purchased from the internet. All four cathinones appeared to be highly pure when checked as described by Apirakkan et al. [6]. HPLC-grade acetonitrile, analytical-grade methanol and formic acid were obtained from Fisher Scientific (Loughborough, UK). Ultra-pure water (18.2 MΩ.cm) was obtained from an Elga Purelab Flex (High Wycombe, UK). Phosphate buffer (0.5 M, pH = 7.4), Ultrapool^™^ human liver microsomes (HLM), NADPH regeneration system solutions A and B, and UDP Reaction Mix Solution A and B were purchased from Corning (Wolburn, MA, USA). 3′-Phosphoadenosine 5′-phosphosulphate (PAPS) was purchased from Merck (Damstadt, Germany).

### 4.2. In Vitro Metabolism Study Using Human Liver Microsomes and S9 Fractions

In vitro experiments were conducted following the protocol published by Menzies et al. [25]. To investigate phase I metabolism, 10 µg of each SCt was mixed in an Eppendorf^®®^ LoBind protein tube (1.5 mL) (Stevenage, UK) with 500 µL of phosphate buffer (0.2 M, pH = 7.4), 50 µL of NADPH-regenerating system A (26 mM NADP^+^, 66 mM glucose-6-phosphate and 66 mM MgCl_2_ in water), 10 µL of NADPH-regenerating system B (40 U/mL glucose-6-phosphate dehydrogenase in 5 mM sodium citrate) and 390 µL of ultra-pure water. The mixture was preincubated at 37 °C for 10 min using an Eppendorf ThermoMixer^®®^ (Stevenage, UK) before 50 µL of HLM (20 mg/mL in 250 mM sucrose) was added to initiate metabolism. The incubation continued for six hours using the Eppendorf ThermoMixer^®®^. After six hours, 50 μL of cold acetonitrile was added to terminate the reaction. The aliquots were centrifuged at 12,000× *g* for 5 min to separate the denatured enzymes and incubates. Phase II metabolism was performed alongside phase I, but instead of adding 390 µL of ultra-pure water, 62.5 µL of UDP reaction mix solution A (25 mM UDP-glucuronic acid in water), 100 µL of UDP solution B (250 mM Tris-HCl, 40 mM MgCl_2_ and 0.125 mg/mL alamethicin in water) and 32.5 µL of deionised water were added to the mixture. Experiments with the S9 fraction containing cytosolic and microsomal enzymes were performed in a similar manner but with the addition of 150 µL of PAPS (1 mg/mL solution in water) and 50 µL of the S9 fraction (20 mg/mL in 250 mM sucrose). Blank reaction mixtures were also run in parallel, comprising all reagents and enzymes as above but without the presence of any drug. Experiments were performed in singlicate. Processed incubates were subsequently injected into the LC–HRMS system. 

### 4.3. LC–HRMS Analysis

LC–HRMS analysis was performed on a Thermo Fisher Scientific Q-Exactive™ Hybrid Quadrupole-Orbitrap™ Mass Spectrometer coupled with Dionex UltiMate 3000 UPLC pump (Hemel Hempstead, UK). A Waters Acquity UPLC^®®^ BEH C18 column (2.1 × 50 mm, 1.7 µm) (Manchester, UK) maintained at 30 °C was utilised for the separation of compounds. The flow rate was 0.3 mL/min, and the aqueous and organic mobile phases used were water (A) and acetonitrile (B), respectively, both containing 0.3% formic acid. The gradient was programmed as follows: 0–0.5 min 5% B, 0.5–3.5 min 20% B, 3.5–5.5 min 25% B, 5.5–7 min 57% B, 7–8 min 90% B, 8–10 min 5% B. The total run time was 13 min, including re-equilibration time. In silico metabolite prediction was performed using ChemDraw 2016 software; predicted metabolites were analysed in positive electrospray ionisation mode (ESI), and the scan method was set to use both full-scan and targeted MS^2^ modes for confirmation. The full-scan method settings were: resolution of 70,000 (FWHM), automatic gain control (AGC) target 1e^6^ and maximum ion injection time 50 ms, scan range *m*/*z* 100–1500. The targeted MS^2^ method settings were: collision energy of 25, resolution of 35,000 (FWHM), AGC target 1e^5^ and maximum injection time 100 ms, isolation window *m*/*z* 1. The data interpretation was performed using Thermo XCalibur^™^ software version 2.2 provided by Thermo Fisher Scientific (Hemel Hempstead, UK).

## 5. Conclusions

The analytical investigation of the in vitro metabolism of four emerging synthetic cathinones (4-MPD, 2-NMC, 4F-PHP and bk-EPDP) resulted in 24 identified and confirmed phase I and II metabolites after six hours of incubation. All metabolites were detected and identified using LC–HRMS. The identification and structure elucidation of these metabolites will facilitate clinical and forensic toxicology investigations of intoxication cases involving their parent compounds.

## Figures and Tables

**Table 1:** Detected phase I and phase II metabolites for 4-MPD, 2-NMC, 4F-PHP and bk-EPDP.

Compound	Proposed Structure	Chemical Formula	Exact Mass [M+H]^+^	Accurate Mass [M+H]^+^	Δ ppm	Rt (min)
**4-MPD**	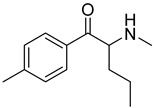	C_13_H_19_NO	206.1539	206.1539	0.0000	5.03
M1	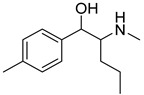	C_13_H_21_NO	208.1696	208.1694	−0.9608	4.89
M2 (a)	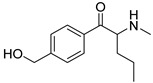	C_13_H_19_NO_2_	222.1489	222.1488	−0.4501	2.62
M3	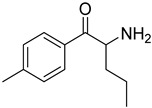	C_12_H_17_NO	192.1383	192.1383	0.0000	4.80
M4	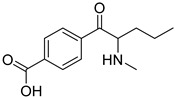	C_13_H_17_NO_3_	236.1281	236.1278	−1.2705	2.44
M5	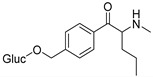	C_19_H_27_NO_8_	398.1809	398.1806	−0.7534	2.24
**2-NMC**	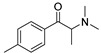	C_12_H_17_NO	192.1383	192.1383	0.0000	3.49
M6 (a)	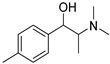	C_12_H_19_NO	194.1539	194.1540	0.5151	3.38
M7	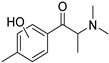	C_12_H_17_NO_2_	208.1332	208.1332	0.0000	4.17
M8	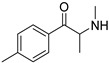	C_11_H_15_NO	178.1226	178.1226	0.0000	3.34
M9	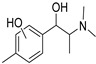	C_12_H_19_NO_2_	210.1489	210.1488	−0.4759	3.55
M10 (b)	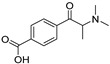	C_12_H_15_NO_3_	222.1125	222.1126	0.4502	1.13
M11	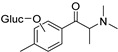	C_18_H_25_NO_8_	384.1653	384.1645	−2.0824	1.03
**4F-PHP**	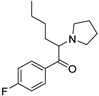	C_16_H_22_FNO	264.1758	264.1754	−1.5141	5.88
M12 (a)	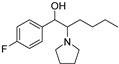	C_16_H_24_FNO	266.1915	266.1911	−1.5027	6.42
M13	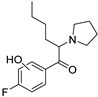	C_16_H_22_FNO_2_	280.1707	280.1703 280.1703	−1.4277 −1.4277	3.39 3.59
M14	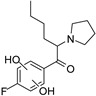	C_16_H_22_FNO_3_	296.1656	296.1652	−1.3506	5.94
M15	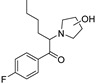	C_16_H_22_FNO_2_	280.1707	280.1703	−1.4277	5.31
M16	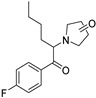	C_16_H_20_FNO_2_	278.1551	278.1547	−1.4380	5.77
M17	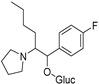	C_22_H_32_FNO_7_	442.2236	442.2234	−0.4523	5.52
M18	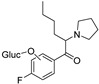	C_22_H_30_FNO_8_	456.2028	456.2026 456.2025	−0.4384 −0.6576	4.67 4.80
**bk-EPDP**	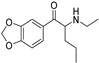	C_14_H_19_NO_3_	250.1438	250.1435	−1.1993	4.38
M19	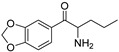	C_12_H_15_NO_3_	222.1125	222.1124	−0.4502	3.95
M20 (a)	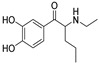	C_13_H_19_NO_3_	238.1438	238.1435	−1.2597	2.48
M21	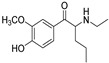	C_14_H_21_NO_3_	252.1594	252.1591 252.1595	−1.1897 0.3966	2.69 4.23
M22	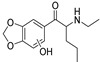	C_14_H_19_NO_4_	266.1387	266.1383 266.1383	−1.5030 −1.5030	3.05 5.27
M23	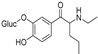	C_19_H_27_NO_9_	414.1759	414.1754	−1.2072	2.06
M24 (b)	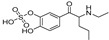	C_13_H_19_NO_6_S	318.1006	318.0997	−2.8293	2.48

(a) Most abundant metabolite; (b) metabolite detected in S9 fraction.

## Data Availability

The data presented in this study are available in this manuscript and supplementary information (https://www.mdpi.com/article/10.3390/metabo12020115/s1).

## Supplementary Materials

The following supporting information can be downloaded at: https://www.mdpi.com/article/10.3390/metabo12020115/s1, Figure S1: Extracted ion chromatogram and MS2 spectra of unmetabolized 4-MPD (proposed fragment ion structures were reported by Apirakkan et al. doi:10.1002/dta.2218), Figure S2: Extracted ion chromatogram and MS2 spectra of 4-MPD M1, Figure S3: 4-MPD M1 proposed fragmentation pattern, Figure S4: 4-MPD M2 MS2 spectra, Figure S5: 2-NMC M6 MS2 spectra, Figure S6: 2-NMC M7 extracted ion chromatogram (Rt = 4.17 min) and MS2 spectra with proposed fragmentation pattern, Figure S7: 2-NMC M9 extracted ion chromatogram (Rt = 3.55 min) and MS2 spectra with proposed fragmentation pattern, Figure S8: Extracted ion chromatogram and MS2 spectra of unmetabolized 4F-PHP (proposed fragment ion structures were reported by Apirakkan et al. doi:10.1002/dta.2218), Table S1: Detected phase I and phase II metabolites for 4-MPD, 2-NMC, 4F-PHP and bk-EPDP including summarised diagnostic product ions as discussed in the paper.
