# Supplementary material for: In Vitro Metabolic Study of Four Synthetic Cathinones: 4-MPD, 2-NMC, 4F-PHP and bk-EPDP
Source: Metabolites. 2022 Jan 25;12(2):115. doi: 10.3390/metabo12020115 (PMC8877857; doi:10.3390/metabo12020115)
Supplement: Supplementary file 1 [file metabolites-12-00115-s001.zip › metabolites-1512269-supplementary.pdf]

Supplementary Materials

In Vitro Metabolic Study of Four Synthetic Cathinones: 4-MPD, 2-NMC, 4F-PHP and bk-EPDP

Ivana Gavrilović [1], Yunita Gelu [2] and Vincenzo Abbate [2,*]

[1] Drug Control Centre, King's College London, Franklin Wilkins Building, 150 Stamford Street, London SE1 9NH, UK; ivana.gavrilovic@kcl.ac.uk
[2] Department of Analytical, Environmental and Forensic Sciences, King's College London, 150 Stamford Street, London SE1 9NH, UK; yunita.gelu@kcl.ac.uk
* Correspondence: vincenzo.abbate@kcl.ac.uk; Tel.: +44-(0)207-83895

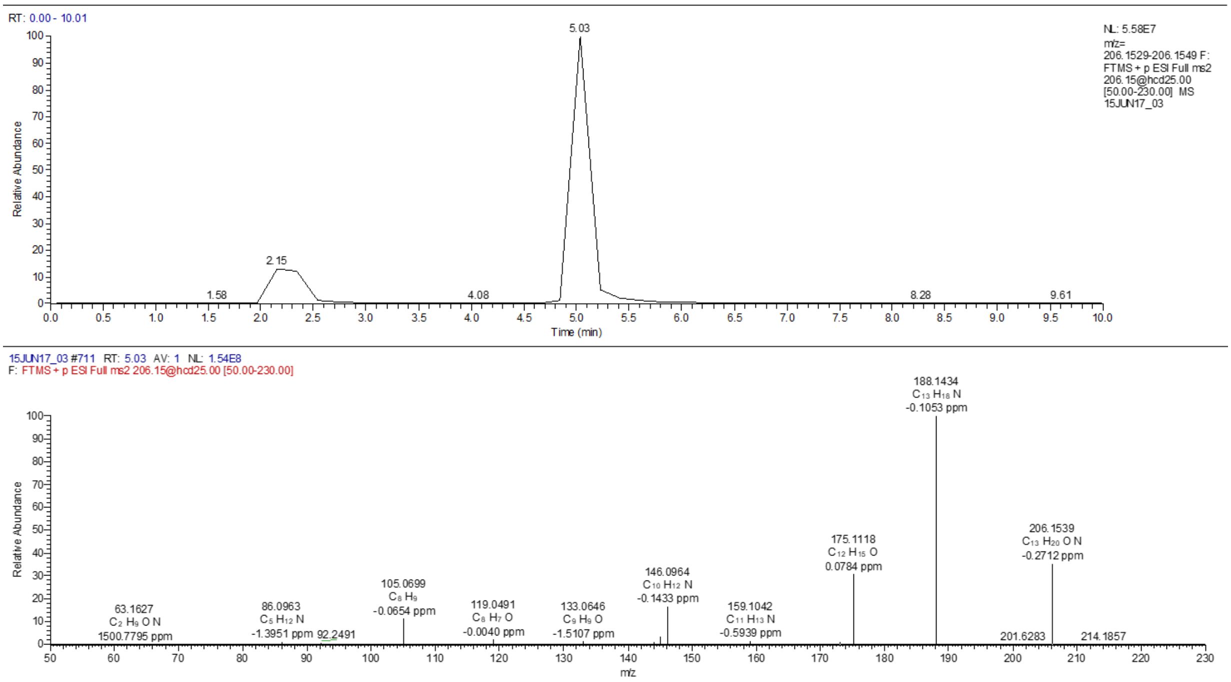

Figure S1: Extracted ion chromatogram and MS² spectra of unmetabolized 4-MPD (proposed fragment ion structures were reported by Apirakkan et al doi:10.1002/dta.2218).

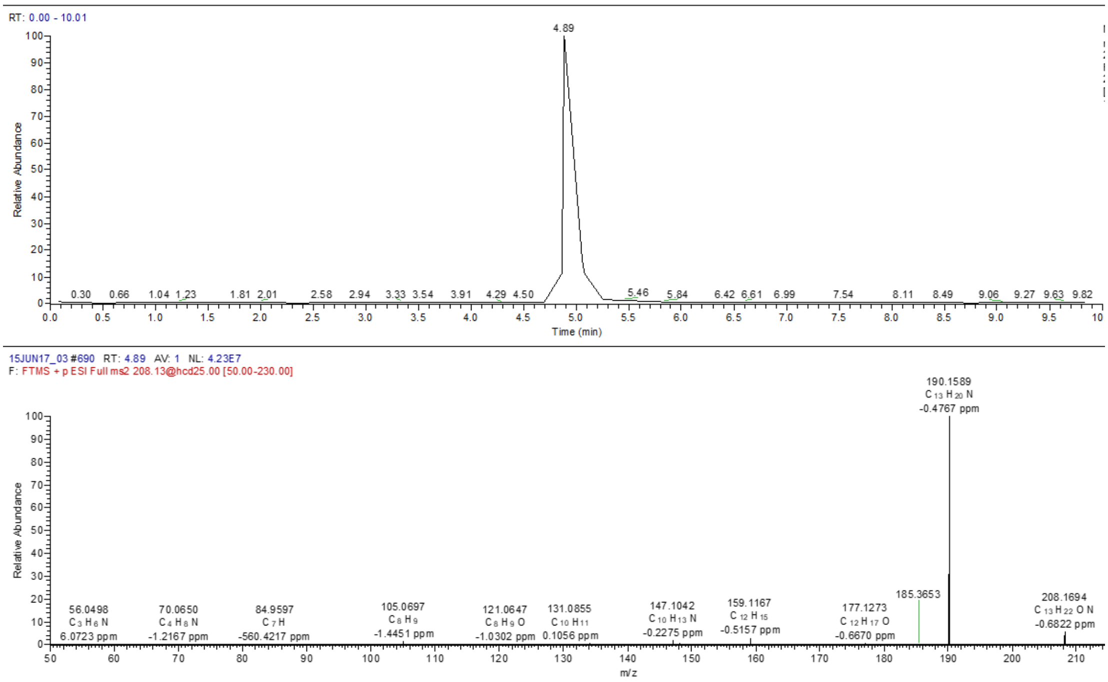

Figure S2: Extracted ion chromatogram and MS² spectra of 4-MPD M1.

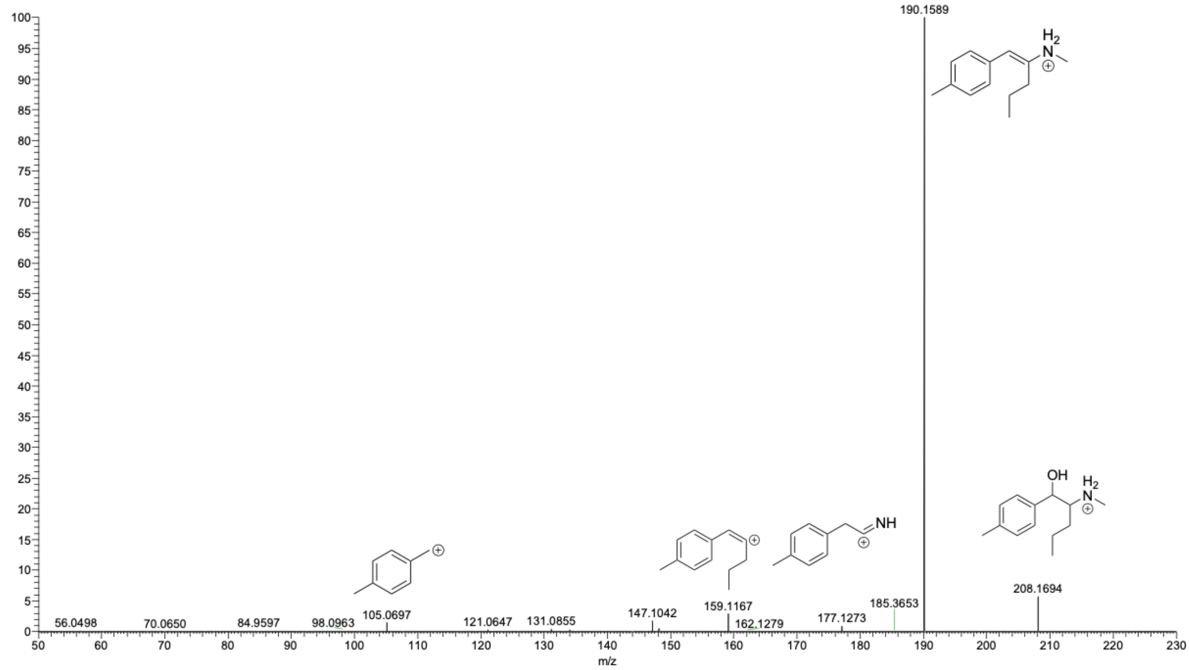

Figure S3: 4-MPD M1 proposed fragmentation pattern.

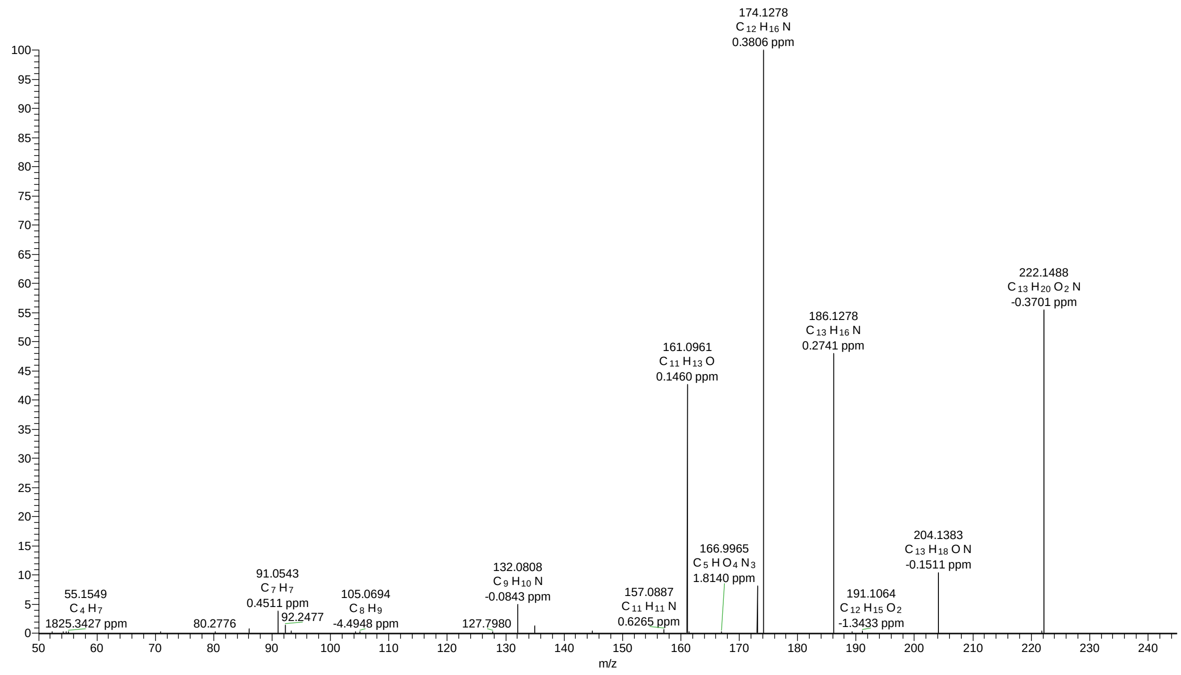

Figure S4: 4-MPD M2 MS² spectra.

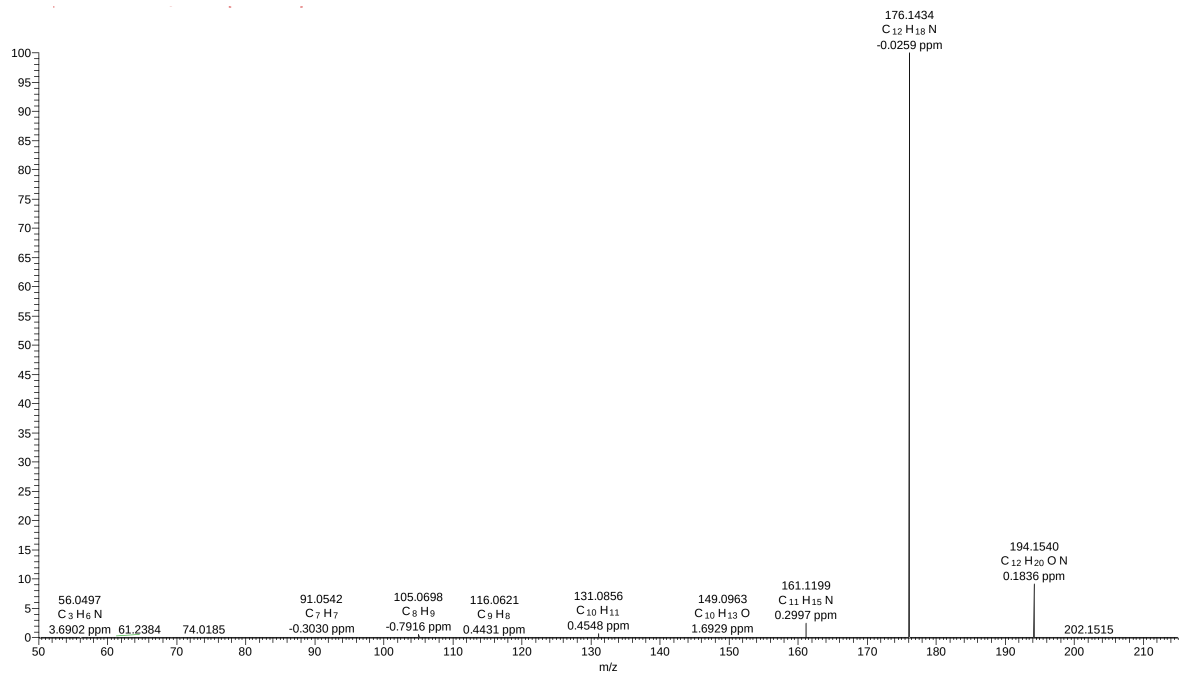

Figure S5: 2-NMC M6 MS² spectra.

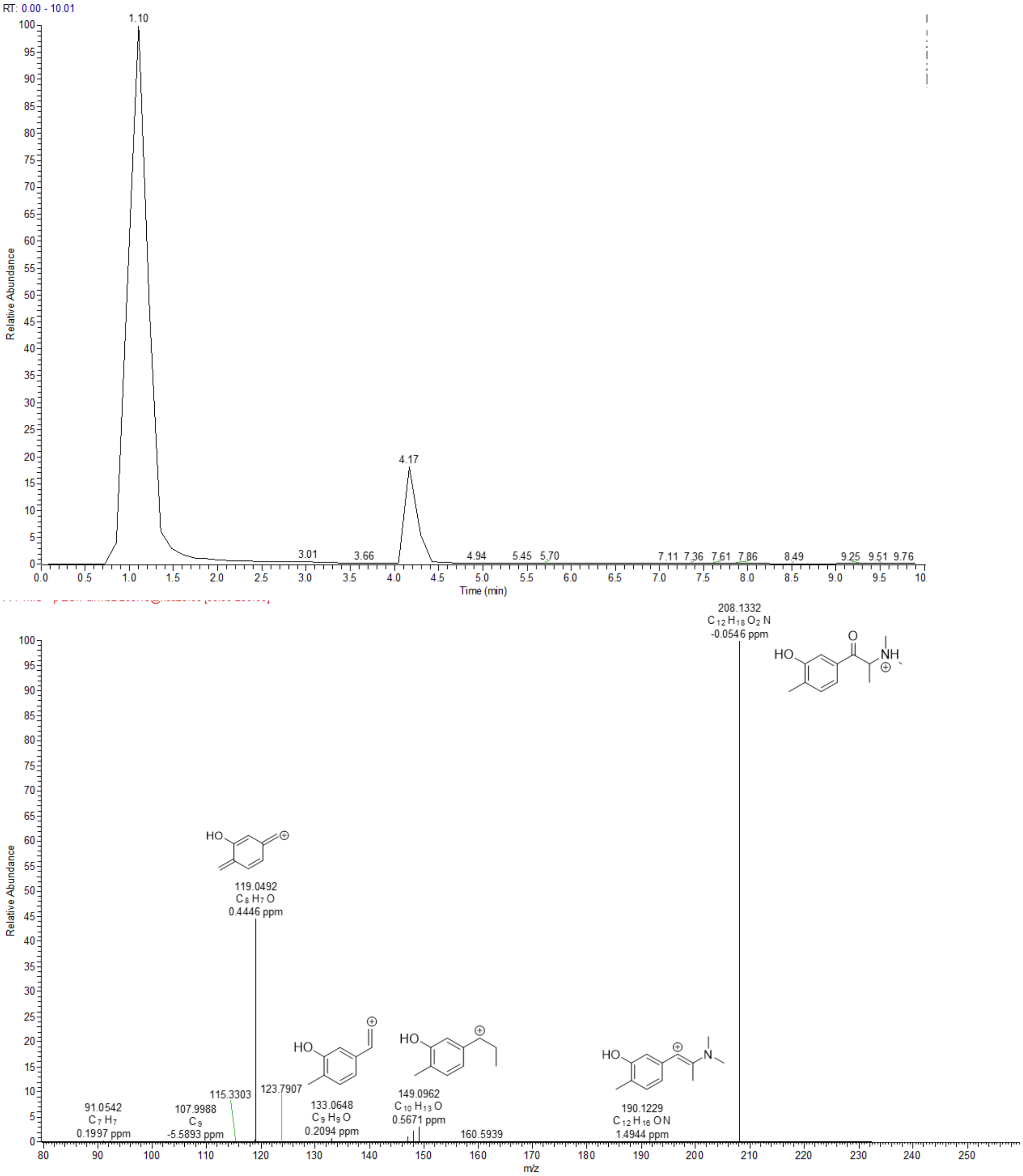

Figure S6: 2-NMC M7 extracted ion chromatogram (Rt = 4.17 min) and MS^2 spectra with proposed fragmentation pattern.

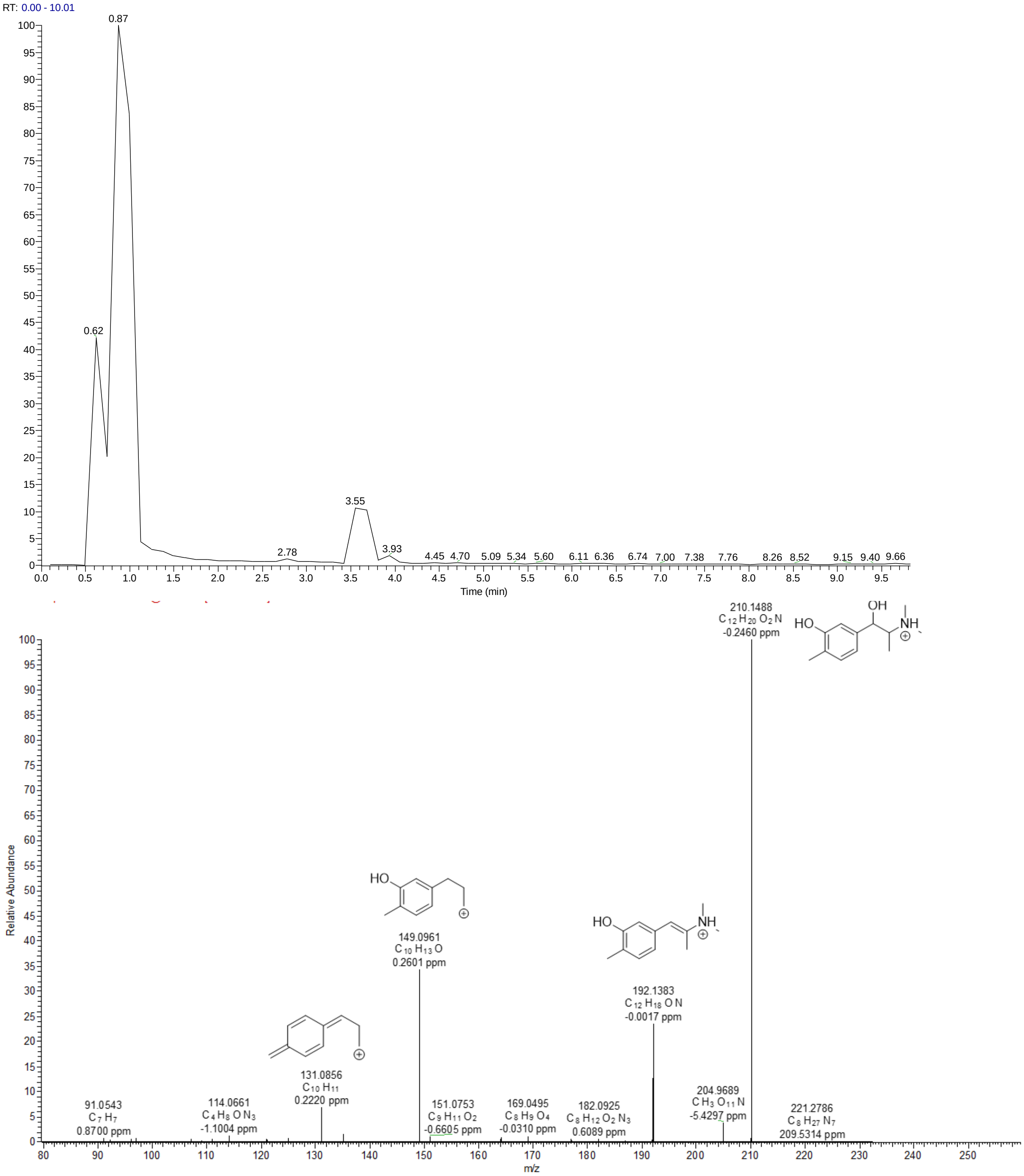

Figure S7: 2-NMC M9 extracted ion chromatogram (Rt = 3.55 min) and MS² spectra with proposed fragmentation pattern.

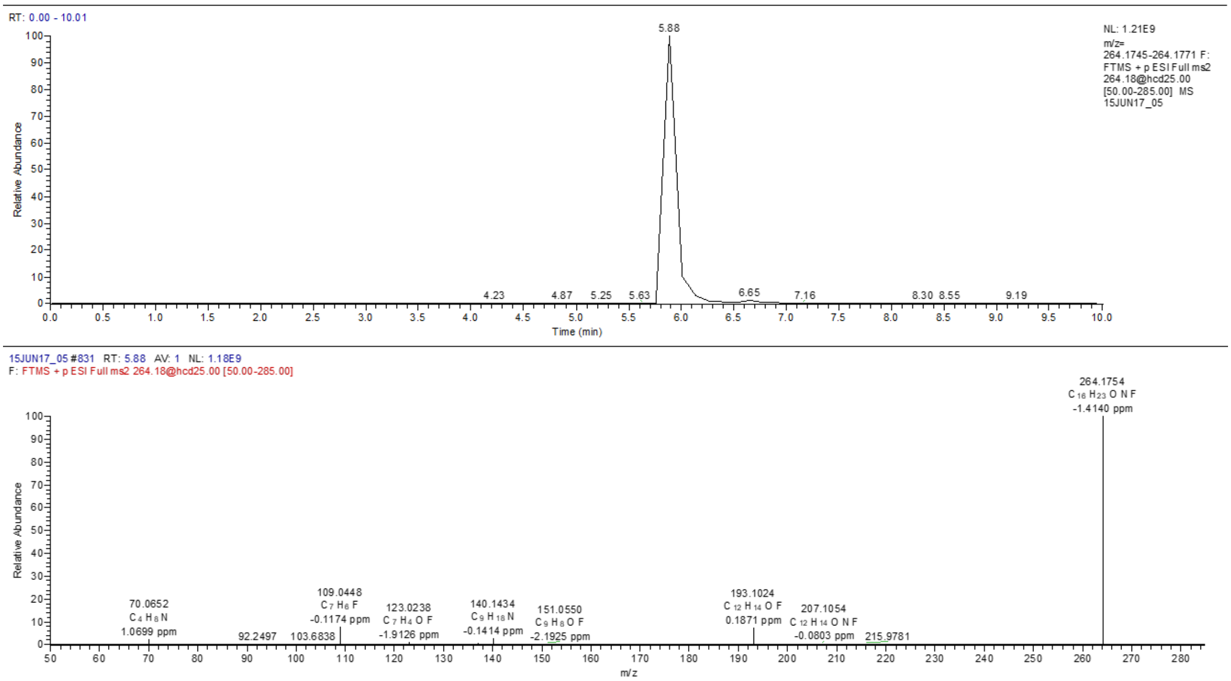

Figure S8: Extracted ion chromatogram and MS² spectra of unmetabolized 4F-PHP (proposed fragment ion structures were reported by Apirakkan et al doi:10.1002/dta.2218).

Table S1: Detected phase I and phase II metabolites for 4-MPD, 2-NMC, 4F-PHP and bk-EPDP including summarised diagnostic product ions as discussed in the paper.

Compound	Proposed structure	Chemical formula	Exact mass [M+H]+	Diagnostic product ions (m/z) with proposed chemical formula	Rt (min)
**4-MPD**	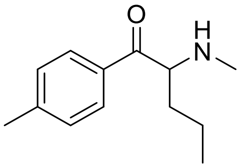	$C_{13}H_{19}NO$	206.1539	206.1539, $C_{13}H_{20}NO$,<br>188.1434, $C_{13}H_{18}N$<br>175.1118, $C_{12}H_{15}O$<br>146.0964, $C_{10}H_{12}N$<br>133.0646, $C_{9}H_{9}O$<br>105.0699, $C_{8}H_{9}$	5.03
M1	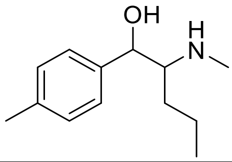	$C_{13}H_{21}NO$	208.1696	208.1694, $C_{13}H_{22}NO$<br>190.1589, $C_{13}H_{20}N$<br>177.1273, $C_{12}H_{17}O$<br>105.0697, $C_{8}H_{9}$	4.89
M2 (a)	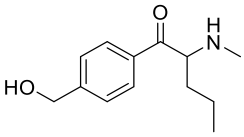	$C_{13}H_{19}NO_2$	222.1489	222.1488, $C_{13}H_{20}NO_2$<br>204.1383, $C_{13}H_{18}NO$<br>186.1278, $C_{13}H_{16}N$<br>174.1278, $C_{12}H_{13}N$	2.62
M3	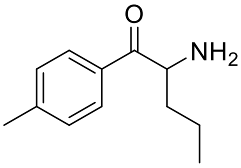	$C_{12}H_{17}NO$	192.1383	192.1383, $C_{12}H_{18}NO$<br>174.1278, $C_{12}H_{16}N$<br>119.0492, $C_{8}H_{7}O$<br>105.0699, $C_{8}H_{9}$	4.80

M4	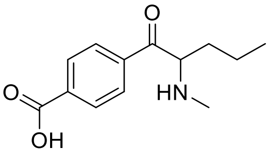	$C_{13}H_{17}NO_3$	236.1281	236.1279, $C_{13}H_{18}NO_3$<br>218.1171, $C_{13}H_{16}NO_2$<br>200.1068, $C_{13}H_{14}NO$<br>174.1275, $C_{12}H_{16}N$	2.44
M5	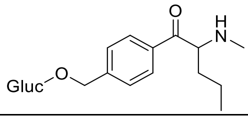	$C_{19}H_{27}NO_8$	398.1809	398.1806, $C_{19}H_{28}NO_8$<br>222.1488, $C_{13}H_{20}NO_2$	2.24
**2-NMC**	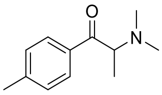	$C_{12}H_{17}NO$	192.1383	192.1388, $C_{12}H_{18}NO$<br>147.0804, $C_{10}H_{11}O$<br>119.0855, C_9H_{11}	3.49
M6 (a)	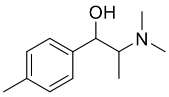	$C_{12}H_{19}NO$	194.1539	194.1540, $C_{12}H_{20}NO$<br>176.1434, $C_{12}H_{18}N$<br>161.1199, $C_{11}H_{15}N$<br>131.0856, $C_{10}H_{11}$<br>105.0698, C_8H_9<br>91.0542, C_7H_7	3.38
M7	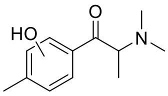	$C_{12}H_{17}NO_2$	208.1332	208.1332, $C_{12}H_{18}NO_2$<br>190.1229, $C_{12}H_{16}NO$<br>149.0962, $C_{10}H_{13}O$<br>133.0648, C_9H_9O<br>119.0492, C_8H_7O	4.17
M8	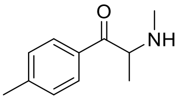	$C_{11}H_{15}NO$	178.1226	178.1226, $C_{11}H_{16}NO$<br>160.1121, $C_{11}H_{14}N$<br>145.0886, $C_{10}H_{11}N$	3.34
M9	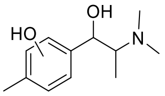	$C_{12}H_{19}NO_2$	210.1489	210.1488, $C_{12}H_{20}NO_2$<br>192.1383, $C_{12}H_{18}NO$<br>149.0961, $C_{10}H_{13}O$<br>131.0856, $C_{10}H_{11}$	3.55
M10 (b)	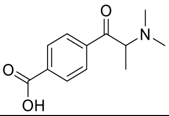	$C_{12}H_{15}NO_3$	222.1125	222.1126, $C_{12}H_{16}NO_3$	1.13
M11	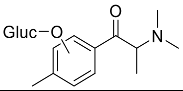	$C_{18}H_{25}NO_8$	384.1653	384.1645, $C_{18}H_{26}NO_8$<br>208.1330, $C_{12}H_{18}NO_2$	1.03
**4F-PHP**	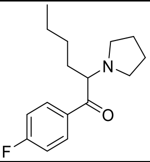	$C_{16}H_{22}FNO$	264.1758	264.1754, $C_{16}H_{23}FNO$<br>193.1024, $C_{12}H_{14}FO$<br>109.0448, C_7H_6F	5.88
M12 (a)	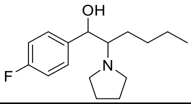	$C_{16}H_{24}FNO$	266.1915	266.1911, $C_{16}H_{25}FNO$<br>248.1806, $C_{16}H_{23}FN$	6.42
M13	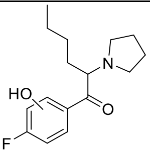	$C_{16}H_{22}FNO_2$	280.1707	280.1703, $C_{16}H_{23}FNO_2$<br>123.0239, C_7H_4FO	3.39<br>3.59
M14	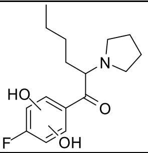	$C_{16}H_{22}FNO_3$	296.1656	296.1652, $C_{16}H_{23}FNO_3$	5.94

M15	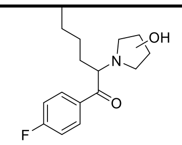	$C_{16}H_{22}FNO_2$	280.1707	280.1703, $C_{16}H_{23}FNO_2$<br>156.1383, $C_9H_{18}NO$	5.31
M16	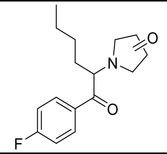	$C_{16}H_{20}FNO_2$	278.1551	278.1548, $C_{16}H_{21}FNO_2$<br>154.1224, $C_9H_{16}NO$	5.77
M17	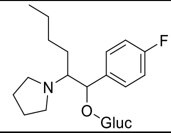	$C_{22}H_{32}FNO_7$	442.2236	442.2234, $C_{22}H_{33}FNO_7$	5.52
M18	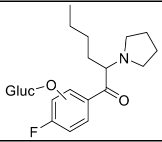	$C_{22}H_{30}FNO_8$	456.2028	456.2025, $C_{22}H_{31}FNO_8$	4.67<br>4.80
**bk-EPDP**	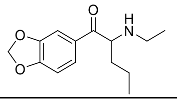	$C_{14}H_{19}NO_3$	250.1438	250.1435, $C_{14}H_{20}NO_3$<br>232.1330, $C_{14}H_{18}NO_2$	4.38
M19	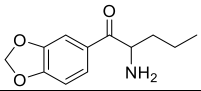	$C_{12}H_{15}NO_3$	222.1125	222.1124, $C_{12}H_{16}NO_3$<br>174.0913, $C_{11}H_{12}NO$	3.95
M20 (a)	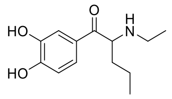	$C_{13}H_{19}NO_3$	238.1438	238.1435, $C_{13}H_{20}NO_3$<br>220.1330, $C_{13}H_{18}NO_2$<br>202.1226, $C_{13}H_{16}NO$<br>193.0859, $C_{11}H_{13}O_3$<br>123.0440, $C_7H_7O_2$	2.48
M21	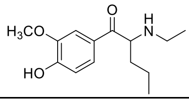	$C_{14}H_{21}NO_3$	252.1594	252.1592, $C_{14}H_{22}NO_3$	2.69<br>4.23
M22	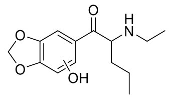	$C_{14}H_{19}NO_4$	266.1387	266. 1384, $C_{14}H_{20}NO_4$	3.05<br>5.27
M23	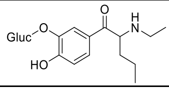	$C_{19}H_{27}NO_9$	414.1759	414.1754, $C_{19}H_{28}NO_9$	2.06
M24 (b)	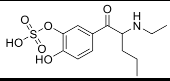	$C_{13}H_{19}NO_6S$	318.1006	318.0997, $C_{13}H_{20}NO_6S$	2.48
